# Fear, Feeding, and the Gut: Nutrition Support Considerations in Adults with ARFID and Gastrointestinal Symptoms

**DOI:** 10.3390/nu18050726

**Published:** 2026-02-24

**Authors:** Jamie Bering, John K. DiBaise

**Affiliations:** Division of Gastroenterology and Hepatology, Mayo Clinic Arizona, 13400 E. Shea Blvd, Scottsdale, AZ 85259, USA

**Keywords:** avoidant/restrictive food intake disorder, disorders of gut-brain interaction, neurogastroenterology, nutrition support therapy

## Abstract

Avoidant/restrictive food intake disorder (ARFID) is an eating disorder characterized by persistent restriction or avoidance of food intake leading to clinically significant nutritional, medical, and/or psychosocial consequences, without associated body-image disturbance. Although historically described in pediatric populations, ARFID is increasingly recognized in adults, particularly among patients with gastrointestinal (GI) disorders. Emerging data demonstrate a strong bidirectional relationship between ARFID and GI disease—especially disorders of gut–brain interaction—where fear of GI symptoms commonly drives restrictive eating, and chronic undernutrition may worsen GI motility, visceral sensitivity, and symptom severity, reinforcing a self-perpetuating cycle. Despite growing recognition of ARFID in adult gastroenterology patients, evidence guiding nutritional management and the use of nutrition support therapies in this population remains limited. This narrative review synthesizes the current literature on the epidemiology, clinical features, and nutritional consequences of ARFID in adults with GI disease, with a focus on screening and diagnostic considerations relevant to GI clinicians and principles of multidisciplinary management. Particular attention is given to the role of nutrition support therapies, including oral nutritional supplementation, enteral nutrition, and parenteral nutrition. While oral strategies are foundational to nutritional rehabilitation, available evidence supporting enteral or parenteral nutrition in adults with ARFID is sparse and largely extrapolated from pediatric or retrospective studies. Expert guidelines caution against routine or prolonged use of invasive nutrition support due to risks of reinforcing food avoidance, medical complications, and poor long-term outcomes, recommending their use only in carefully selected, medically necessary, and time-limited circumstances. Overall, ARFID represents an underrecognized but clinically significant contributor to malnutrition and symptom burden in adult patients with GI disorders, underscoring the need for routine screening, individualized multidisciplinary care, and high-quality prospective research to inform evidence-based treatment guidelines.

## 1. Introduction

Avoidant/restrictive food intake disorder (ARFID) is an eating disorder characterized by the persistent avoidance or restriction of food intake that leads to one or more clinically significant consequences, including weight loss, nutritional deficiency, dependence on nutritional support therapies, and/or marked psychosocial interference [1]. Unlike other eating disorders, ARFID is distinct in that the associated disordered eating patterns are not driven by body-weight or body-image concerns. The eating habits of patients with ARFID are often highly selective and driven by sensory sensitivities, such as aversions to certain food textures, etc., rather than by a preoccupation with body weight or shape. Diagnostic features span three presentations that can co-occur: (1) avoidance based on sensory characteristics of food; (2) apparent lack of interest in eating/low appetite; and (3) avoidance due to fear of aversive consequences (e.g., choking, vomiting, or pain). These criteria were clarified in the American Psychiatric Association (APA) DSM-5 Text Revision (DSM-5-TR) to improve clinical specificity [1] (Table 1).

While originally described in the pediatric population, ARFID is being described more commonly in adults. Estimates of ARFID prevalence vary. In children and adolescents, ARFID prevalence is estimated to be between 0.3% and 15.5%, and may be more common among certain populations, such as those with psychiatric comorbidities like autism spectrum disorders or anxiety [2]. Among adults, the data remain limited but suggest that prevalence in the general population ranges from 0.3% to 5%, while some clinical series report up to 11% prevalence among samples that include patients with eating disorders [3,4].

The intersection of ARFID with gastrointestinal (GI) symptoms is clinically salient. Fear of GI symptoms is a common motivator for restrictive eating, and adults referred for gastroenterology evaluation may demonstrate a substantial burden of ARFID symptoms. In one tertiary-care gastroenterology practice, a retrospective review of 410 consecutive adult referrals (ages 18–90; 73% female) for neurogastroenterology evaluation revealed that 6.3% of patients met full ARFID criteria and an additional 17.3% had clinically significant avoidant/restrictive eating, yielding 23.6% with ARFID-spectrum presentations. Among those with ARFID symptoms, 92.8% endorsed fear of GI symptoms as a primary motivator for restriction [5]. Another population-based study of Swedish adults aged 18–70 years showed that avoidant/restrictive eating was more common in individuals with bowel symptoms (*n* = 825) compared to controls (*n* = 1806) (22.8% vs. 18.2%, *p* < 0.001) [6]. Individuals in this study with bowel symptoms and avoidant/restrictive eating were more often female, had lower body mass index, were more likely to report overlapping functional dyspepsia, and reported more severe bowel, psychological, and somatic symptoms, shape/weight concerns, and a lower quality of life. Furthermore, bowel symptom severity emerged as the strongest factor explaining the variability in avoidant/restrictive eating severity. These findings highlight bidirectional links between disorders of gut–brain interaction (DGBI) and ARFID, and emphasize the potential importance of routine ARFID screening in GI practice [7]. While prevalence estimates vary, the data would suggest that ARFID is being increasingly recognized as a common comorbidity in adult gastroenterology practices, particularly among patients with disorders of gut–brain interaction (DGBI) [7,8,9]. The recent literature also highlights a surge in interest and recognition of ARFID in GI settings, especially as clinicians become more attuned to eating disorders not centered on body-image concerns [7,8,10].

Despite this overlap, evidence to guide nutrition support therapy (i.e., oral nutritional supplements, enteral nutrition, and parenteral nutrition) in adults with ARFID is sparse. Systematic reviews note few interventional studies, an absence of standardized nutrition protocols, and the literature is dominated by pediatric cohorts and retrospective case series [11]. When nutrition support is used, recommendations stress individualized, multidisciplinary care with priority on oral refeeding and cautious, time-limited use of tube feeding when medically necessary [12]. Overall, high-quality trials in adults are lacking, and recommendations generally rely upon expert opinion. This review, therefore, synthesizes the available evidence and pragmatic approaches to nutrition support therapy for adults with ARFID, focusing on those presenting with concomitant GI disorders, to help inform clinical decision-making and define research priorities.

This narrative review is based on a non-systematic literature search. A systematic review was not pursued due to the limited number of high-quality, comparable studies currently available in this area. Relevant articles were identified through searches in the PubMed database using the keywords ARFID, avoidant restrictive food intake disorder, neurogastroenterology, disorder of the gut–brain axis, eating disorder, nutrition support, enteral nutrition, and parenteral nutrition. The search was limited to English-language publications from the last 10 years (2015–2025), although key earlier studies were included if considered foundational. Additional articles were identified by screening the reference lists of included publications.

## 2. Overview of ARFID

As previously described, the APA defines ARFID as avoidant or restrictive eating behaviors that result in clinically significant nutritional deficiency or functional impairments [1]. Importantly, the disturbance is not attributable to other factors such as lack of available food, culturally sanctioned practices, or another medical or psychiatric disorder, nor is it associated with distorted body image [1,12]. Three main subtypes of ARFID are recognized: (1) sensory-based avoidance, (2) fear of aversive consequences, and (3) lack of interest in eating or food [1,12]. Sensory-based avoidance is typified by limited eating due to the sensory characteristics of food like texture, taste, and smell. The subtype relating to fear of aversive consequences is characterized by restricted eating due to fear of negative outcomes like GI symptoms, such as abdominal discomfort, choking, nausea, or vomiting. Finally, the third subtype is distinguished by an apparent indifference to food or eating.

ARFID is most commonly identified in children and adolescents, but can also present in adults. The American Academy of Pediatrics notes that ARFID was previously limited to childhood but now encompasses all ages. Pediatric presentations often involve the failure to gain weight or grow as expected, while adults may present with weight loss, chronic nutritional deficiencies, or psychosocial impairment [1,13]. In adults, clinically significant weight loss is defined as a 5% unintentional weight loss in 1 month, or a 7.5% loss over 3 months [14]. Weight loss can contribute to nutritional deficiency and malnutrition, although nutritional deficiency can occur even when individuals are eating a sufficient amount of food due to limited variety of foods in the diet. Notably, while malnutrition is not required for ARFID diagnosis, it is common in severe cases [1,13].

## 3. ARFID in Adults with GI Disorders

Adult patients with ARFID commonly present with overlapping GI symptoms or disorders, including DGBI such as irritable bowel syndrome (IBS) or functional dyspepsia (FD), and other GI disorders like gastroparesis, celiac disease, eosinophilic esophagitis, achalasia, and inflammatory bowel disease [15]. Commonly reported GI symptoms include dysphagia, nausea, vomiting, abdominal pain, bloating, and altered bowel habits [16]. In one study of hospitalized adults with underweight ARFID, 64% had a history of three or more GI diagnoses, including DGBI, and 100% presented with a minimum of five GI symptoms [17]. Individuals may initially develop food avoidance due to GI symptoms, and this can develop into a chronic condition that may persist even after resolution of the initial insult or underlying GI condition [18].

In addition, psychiatric comorbidities are common in adults with ARFID and can play a critical bidirectional role in symptom perpetuation, particularly in patients with overlapping gastrointestinal disorders. Anxiety disorders are the most frequently reported psychiatric overlap and may both precede or arise as a consequence of restrictive eating [19]. Heightened anxiety can amplify visceral hypersensitivity, GI-specific hypervigilance, and fear of aversive consequences, thereby reinforcing food avoidance [20]. Depression is also prevalent in adults with ARFID and may contribute to reduced appetite, lowered motivation to eat, and impaired engagement in treatment. Depressive symptoms may worsen with dietary restriction, contributing to a self-reinforcing cycle [21].

The prevalence of ARFID symptoms among adults seen in neurogastroenterology and DGBI clinics ranges from 6 to 40% [5,7,22,23]. As such, it is important for clinicians to be aware of ARFID, when to suspect it, and how to diagnose it. Screening questionnaires, such as the Nine-Item ARFID Screen (NIAS), can be used to help clinicians identify patients who may be suffering from ARFID. The NIAS is a validated screening tool consisting of nine questions composed of three subscales—picky eating, loss of appetite, and fear—each representing the different presentations of ARFID [24,25]. It has been used in various populations, including adults, and is viewed as a quick way to identify potentially problematic eating behaviors [24,25]. The NIAS has not been validated in those with chronic GI disorders, and this is an area of future research. Importantly, those who screen positive for ARFID on the NIAS require a formal diagnostic assessment to confirm the diagnosis.

When a patient screens positive on the NIAS, the APA recommends a comprehensive initial psychiatric evaluation, including specific assessments of eating patterns, weight history, and psychosocial functioning [12]. For ARFID specifically, a development history of feeding and eating is particularly important, exploring early feeding problems, oral-motor problems, and food intolerances or allergies that may have contributed to aversive conditioning. Medical conditions, such as gastroesophageal reflux disease (GERD), eosinophilic esophagitis, and inflammatory bowel disease, should be evaluated as potential contributors [12]. For patients with confirmed ARFID, referral to an interdisciplinary eating disorder team is recommended as the multidisciplinary approach addresses the dual goals of normalizing eating patterns while simultaneously managing any underlying comorbidities.

Although the NIAS provides a time-efficient method for identifying ARFID behaviors in clinical practice, it has not been formally validated in populations with chronic gastrointestinal disorders. This limitation has important implications for clinical application in GI settings where symptom overlap is common. As such, NIAS results in GI populations should be interpreted with caution and as a prompt for further clinical inquiry and evaluation, rather than a stand-alone diagnostic indicator. Future studies validating ARFID screening tools in patients with GI symptom overlap are needed.

A recent scoping review evaluating ARFID in the context of neurogastroenterology reported a wide range of GI disorders and related GI symptoms among patients with ARFID [26]. In patient samples presenting with gastroparesis symptoms, 40% met NIAS cutoffs for ARFID symptoms, but only approximately 23% had “definite ARFID” (i.e., with documented medical or psychosocial impairment). Among adult outpatients with ARFID, the frequency of functional dyspepsia, IBS, gastroparesis, GERD, and chronic constipation was notably high: IBS in 28.8%, GERD in 13.4%, gastroparesis in 12.4%, and chronic constipation in 34%. These data suggest that ARFID in adults frequently coexists with a spectrum of GI disorders, complicating both diagnosis and management.

Flack et al. conducted a population-based survey that incorporated use of the Rome IV diagnostic questionnaire for DGBI and the NIAS, among other questionnaires, and found that the prevalence of ARFID-positive screens was significantly higher among participants with DGBI compared with those without DGBI (34.6% vs. 19.4%; adjusted odds ratio, 1.67; 95% confidence interval, 1.43–1.94) [20]. Furthermore, they found that individuals with DGBI plus ARFID, compared with those with DGBI alone, were significantly more likely to be underweight (7.9% vs. 1.5%), have greater non-GI somatic symptoms and psychological distress, have reduced mental and physical quality of life, and display increased healthcare use [20].

Although most of the published data come from patients with defined DGBI, motility disorders, or structural GI disease, the pathophysiological mechanisms (e.g., GI dysmotility, altered gut–brain signaling, or impaired gut barrier/integrity) apply to a broader set of conditions. For instance, patients with postural orthostatic tachycardia syndrome (POTS), other forms of dysautonomia, joint hypermobility syndromes, and mast-cell activation disorders frequently report GI symptoms (e.g., nausea, early satiety, bloating, or constipation) despite the absence of overt structural abnormalities—a clinical scenario analogous to functional/motility GI disorders [27]. While direct data on ARFID in such populations are limited, the plausible overlap suggests these patients may be at risk for restrictive eating behaviors, malnutrition, and nutrient deficiencies. Indeed, a recent review on the overlap between eating disorders and GI disease argues for thorough investigation of GI disorders in patients with eating disorders, and conversely for screening for disordered eating in individuals with unexplained GI complaints, even when a clear diagnosis is not established [28]. Given the high prevalence of non-specific GI symptoms in patients with autonomic or motility disorders and their impact on nutrition, this gap represents an important underexplored area for future research.

Autonomic dysfunction and intestinal motility disorders represent clinically important and increasingly recognized areas of overlap with ARFID in adults. Conditions such as POTS, dysautonomia, gastroparesis, and functional motility disorders are frequently associated with symptoms—including early satiety, nausea, bloating, abdominal pain, constipation, and postprandial fatigue—all of which may precipitate or perpetuate restrictive eating behaviors [27]. Altered autonomic regulation and dysregulated gut–brain signaling may contribute to both symptom burden and symptom hypervigilance, reinforcing fear-based avoidance of eating [4,5]. In some patients, dietary restriction initially develops as an adaptive response to symptoms that can evolve into maladaptive patterns consistent with ARFID [7,20]. Conversely, prolonged restriction and malnutrition may further impair gastrointestinal motility and autonomic function, creating a self-perpetuating cycle of symptoms and avoidance [18]. Recognition of this overlap is critical to clinical management, as an over-attribution of symptoms to “intestinal failure” may increase the risk of unnecessary invasive nutrition support and delay appropriate multidisciplinary intervention.

While patients may initially develop ARFID related to eating-related aggravation/precipitation of GI symptoms, the chronic restrictive eating patterns seen in ARFID can also lead to or exacerbate GI symptoms. This can ultimately lead to a self-perpetuating cycle where restrictive eating results in malnutrition. Importantly, and further complicating the picture, malnutrition can induce or exacerbate GI dysfunction through mechanisms such as delayed gastric emptying, altered intestinal motility, and/or visceral hypersensitivity [7]. These symptoms may further reinforce avoidance of eating, and the cycle continues. Consequently, it is suggested that routine screening for ARFID be performed in individuals presenting for evaluation of DGBI, as early identification can help facilitate timely intervention from a multidisciplinary team [20]. Screening tools, like the NIAS, can also be used to start a conversation with patients about their eating and relationship with food [20,24]. Finally, consideration for dietitian referral should be made early in the evaluation course of these patients, as dietitians can further help with screening for disordered eating, provide comprehensive nutritional assessments, and help focus on the nutritional rehabilitation of the patient [29].

### Case Presentations

To further illustrate the relationship between GI illness and ARFID, two hypothetical patient cases are described. First, consider a 28-year-old man with newly diagnosed eosinophilic esophagitis. Symptoms on initial presentation consisted of dysphagia and an episode of food impaction requiring endoscopic intervention for removal. After being diagnosed with eosinophilic esophagitis, he began to avoid many food groups out of fear of food getting “stuck”. He restricted his intake to include only soft foods such as mashed potatoes, applesauce, and protein shakes. He subsequently reported a 15-pound weight loss and reduced participation in social activities that involved eating. His primary GI condition, eosinophilic esophagitis, started the pattern of restrictive eating, but his avoidance behaviors escalated beyond medical necessity and became maladaptive, meeting ARFID criteria.

In this case, the patient meets ARFID criteria based on his clinically significant weight loss and progressive dietary restriction beyond medical necessity with associated psychosocial impairment. Although eosinophilic esophagitis initially precipitated food avoidance, the persistence and escalation of restriction despite the initiation of disease-directed therapy support a diagnosis of ARFID rather than adaptive symptom management alone. Management decisions should, therefore, prioritize both treatment of the underlying esophageal disease and targeted intervention for ARFID. Nutritional support is justified to address weight loss and prevent nutritional deficiencies, with an emphasis on oral nutrition using texture-modified but nutritionally complete foods and oral supplements rather than enteral feeding, given preserved swallowing function and GI tract integrity. Early involvement of a dietitian and behavioral health provider is essential to facilitate gradual diet expansion and reduce fear-based avoidance.

The second case illustrates the cyclical reinforcement between ARFID and GI symptom overlap. A 35-year-old woman with IBS is seen in the GI clinic where she reports recurrent abdominal pain and diarrhea, especially after meals. Over time, to control her symptoms, she self-restricted her diet to a handful of “safe” foods. Despite initial improvement in symptoms, with continued progressive diet restriction, she developed weight loss with associated symptoms of fatigue and recurrent flares of her IBS. As can be seen, this patient’s initial GI disorder led to avoidance of food due to fear of symptoms, ultimately progressing into ARFID. The limited diet then resulted in malnutrition with perpetuated IBS flares, reinforcing the fear–avoidance cycle.

Together, these cases highlight how ARFID and GI conditions can mutually reinforce each other. ARFID can lead to nutritional deficiencies, weight loss/malnutrition, and GI dysfunction, while GI disorders can lead to fear of food and the development of ARFID. Together, they can progress into a vicious cycle of worsening restriction, symptoms, and malnutrition.

This case illustrates a common presentation in which restrictive eating initially improves GI symptoms, but subsequently can become maladaptive. This patient meets ARFID diagnostic criteria due to sustained restricted eating resulting in weight loss, nutritional compromise, and symptom perpetuation despite the absence of progressive structural disease. Management decisions, therefore, should focus on interrupting the cycle of avoidance and symptom amplification through combined GI symptom management and nutritional rehabilitation. Nutritional support is justified to restore caloric adequacy and prevent further nutritional decline, with careful avoidance of prolonged elimination diets that may reinforce restrictive behaviors. Rather than escalation to enteral nutrition, which may reinforce avoidance, initial treatment should emphasize symptom-directed pharmacotherapy, dietitian-guided food reintroduction, and gut–brain behavioral therapy to address fear symptom exacerbation and improve tolerance of oral intake.

Together, these cases demonstrate how application of ARFID diagnostic criteria, careful assessment of nutritional severity, and selection of dietary support strategies can guide individualized management while minimizing the risk of iatrogenic harm.

## 4. Nutritional Consequences of ARFID with GI Disease

Adults with overlapping ARFID and GI disorders/symptoms can develop nutritional deficits due to both behavioral restriction and disease-driven dietary limitations. ARFID poses a significant impact on the variety, quality, and quantity of the diet, and is associated with an inadequate composition of dietary macronutrients and micronutrients [30]. These patients generally consume reduced total energy, commonly engaging in a diet consisting of higher amounts of processed and refined carbohydrates, foods with added sugars, and lower amounts of protein, fruits, and vegetables [30]. Due to limited dietary variety and avoidance of nutrient-rich foods, patients with ARFID can develop vitamin and mineral deficiencies, particularly vitamin D, vitamin B12, potassium, and iron [15,20,31]. Lim et al. have shown that in patients with inflammatory bowel disease participating in dietary exclusion, the average daily intake of calcium, vitamin A, and zinc was significantly lower compared to those who did not exclude foods [32]. Malabsorption may also be present in GI disorders and can further exacerbate this problem [15,20,31].

Additional nutrition-related complications that may occur in this population include sarcopenia and metabolic bone disease. Sarcopenia, loss of muscle mass and strength, is a significant concern in adults as it can increase the risk of frailty and lead to other adverse clinical outcomes over time [18,31]. Low bone mineral density has also been observed in this population and can occur in part due to deficiencies in calcium and vitamin D, low protein intake, and chronic undernutrition [18,33]. The American Psychiatric Association recommends regular assessment of bone health in patients with ARFID, especially when GI comorbidities are present [12].

Given these risks, routine nutritional monitoring is an important component of long-term care. It is important to acknowledge, however, that ARFID-specific monitoring guidelines are lacking. Baseline laboratory testing commonly includes complete blood count, comprehensive metabolic panel, iron studies, vitamin B12, folate, and vitamin D, along with additional testing guided by dietary patterns and clinical features. Laboratory assessment could reasonably be repeated at 3–6-month intervals during times of active restriction, weight loss, or to follow replacement of identified deficiencies. Less frequent monitoring, such as annually, could be followed once nutritional intake and weight have stabilized.

## 5. Principles of Treatment in ARFID-Positive GI Disease

**Figure 1:**
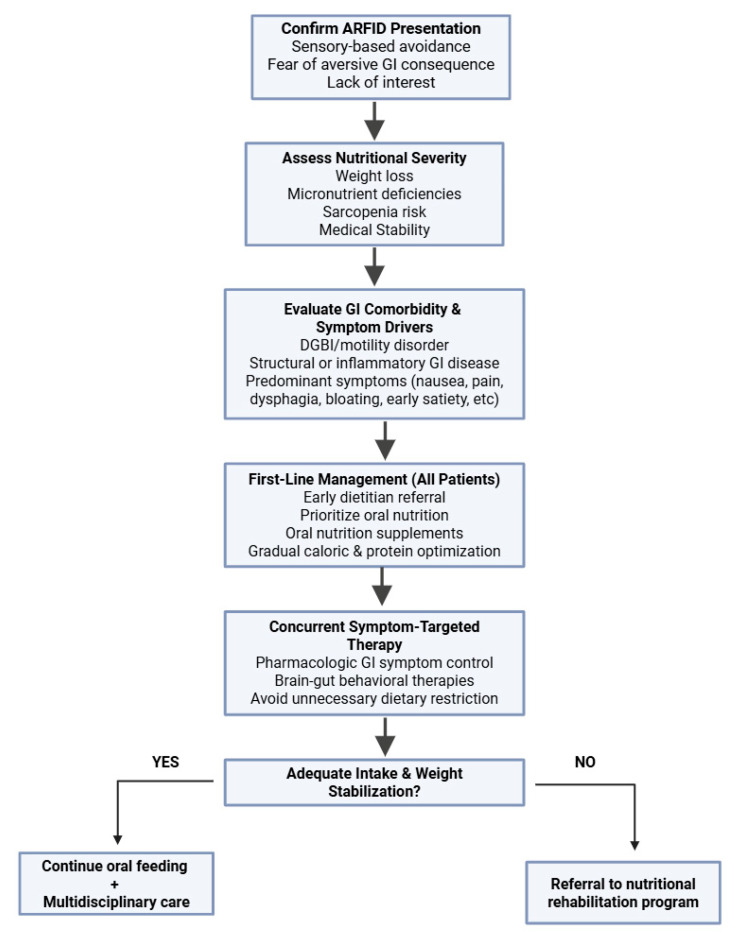
Suggested Treatment Algorithm for Patients with ARFID and GI Symptom Overlap.

Although standardized treatment protocols for ARFID in adult patients have not yet been established, expert reviews and guidelines consistently emphasize that ARFID management is best provided by an individualized, multidisciplinary approach due to the complex interplay of medical, nutritional, and psychological factors [34,35,36]. These teams should include mental and physical healthcare professionals, including physicians, dietitians, eating disorder specialists, psychologists, social workers, and nurse specialists, among others. A suggested treatment algorithm is provided in Figure 1.

Gastroenterologists play an important role in successful ARFID treatment by managing the GI symptoms while avoiding overly restrictive diets [8]. As previously noted, GI symptoms can perpetuate and exacerbate ARFID. Fear of aversive consequences from eating—including abdominal pain, nausea, and vomiting—constitutes a primary driver of food avoidance in ARFID [1,7,37]. Pharmacotherapy typically begins with standard symptomatic measures (Table 2) [7,8,38,39,40,41,42]. When unsuccessful or incomplete, other treatment options include the use of neuromodulators. Olanzapine has shown particular promise given its efficacy in reducing chronic nausea and vomiting and promoting weight gain in eating disorders [8]. Gut–brain behavioral therapies, such as gut-directed hypnosis or cognitive behavioral therapy, may complement ARFID-focused interventions [8]. Importantly, care should be taken to monitor for markers of problematic restriction, including weight loss, inadequate energy intake, psychological distress around eating, or inflexible attitudes toward dietary expansion [8].

In addition to pharmacotherapy, therapeutic diets, such as the low-FODMAP diet, may be considered for symptom management for some patients. It is important to consider, however, that elimination diets in this population may inadvertently reinforce fear-based avoidance, rigidity around food choices, and perceptions of food as inherently harmful, and may lead to exacerbation of ARFID symptoms. As such, these should be considered with caution and under the guidance of trained GI dietitian supervision and discontinued if there is a lack of symptom improvement [7,8].

In regard to treatments specifically addressing ARFID, Hellner et al. show that patients who receive cognitive behavioral therapy (CBT) for ARFID or family based treatment enhanced by support from a multidisciplinary team demonstrated reliable symptom improvement over the course of treatment across all measures [43]. Other studies have shown that dietitians and behavioral health providers can effectively deliver CBT for ARFID as part of a multidisciplinary team, resulting in meaningful clinical improvements [44,45]. Inpatient multidisciplinary models have also shown high rates of treatment goal achievement and sustained progress in adults with ARFID when individualized behavioral therapy is combined with medical oversight [46]. It is acknowledged that these studies are limited by their small patient populations, and larger trials are needed to confirm these findings. Furthermore, while CBT and gut-directed therapies show promise, access may be limited by the availability of trained providers and health insurance coverage. Telehealth delivery and the integration of behavioral health within GI practices may be practical strategies to improve access and reduce treatment gaps.

Nutritional rehabilitation for patients with ARFID should be aimed at restoring nutritional adequacy, correcting deficiencies, and normalizing eating patterns. The APA recommends that nutritional rehabilitation for ARFID patients prioritize medical stabilization, weight restoration if needed, and correction of biological and psychological sequelae of malnutrition, if any, paralleling approaches used for treating other restrictive eating disorders [12]. The Academy of Nutrition and Dietetics further emphasizes the importance of a comprehensive assessment, including anthropometrics, laboratory studies, and dietary history, to help guide intervention [29].

The clinical features and management challenges of ARFID in adults with overlapping GI disorders also align closely with contemporary psychogastroenterology frameworks that emphasize the bidirectional interactions between psychological processes, central nervous system function, and gastrointestinal physiology [47]. Recent psychogastroenterology reviews highlight the importance of integrated, interdisciplinary care models that combine GI symptom management, nutritional rehabilitation, and targeted behavior interventions to address maladaptive gut–brain interactions and illness-related fear or hypervigilance [47,48,49,50]. Applying these principles to ARFID supports early collaboration among gastroenterologists, dietitians, and mental health providers, and reinforces the need for coordinated treatment strategies rather than isolated symptom-focused care.

## 6. Nutrition Support Therapies: Modalities and Applications

### 6.1. Oral Nutrition Support

The approach to nutrition management in ARFID begins with nutritional counseling, use of modified therapeutic diets tailored to the patient’s sensory and psychological tolerances, and oral nutrition supplements [51]. Current recommendations suggest that initial efforts to provide nutritional supplementation should focus on increasing preferred foods and providing additional calories, using oral nutritional supplements when necessary [52,53]. As noted previously, cognitive behavioral therapy may result in significant improvements in diet variety, nutritional status, and ARFID symptom severity in adults, including those with comorbid gastrointestinal disorders [36,43,44,54].

### 6.2. Alternative Nutrition Support Therapies

When patients are unable to meet their nutritional needs with oral supplementation, despite the above measures, or when they are severely malnourished, alternative nutrition support options may need to be considered. However, guidelines regarding the use of nutrition support therapies—including enteral nutrition (EN) and parenteral nutrition (PN)—in adult patients with ARFID are lacking.

Guidance specific to EN use in adult patients with ARFID is limited and based on expert opinion. The APA clinical guidelines for eating disorders note that several factors should be taken into account prior to initiating EN in these patients, including their medical stability, degree of malnutrition, age, and access to specialized treatments (GRADE 1C recommendations) [12]. Potential benefits of EN use in the treatment of ARFID include the potential for rapid caloric restoration, especially in severely malnourished or medically unstable patients. Importantly, the APA notes that while use of EN via nasogastric tube can help restore weight, it has minimal impact on the normalization of food intake or increasing diet variety, and is associated with risks of complications, dependence, and continued poor oral intake. EN can inadvertently reinforce avoidance of oral feeding, especially in individuals with fear-based ARFID subtypes [55]. There is also the risk of complications related to EN administration, including tube-related discomfort, gastroesophageal reflux, and epistaxis with nasoenteric tube use [52,56]. Consequently, the APA suggests that EN should be regarded as a temporary intervention only [12].

Contrary to enteral nutrition, the clinical guidance regarding use of parenteral nutrition in this population is clearer. According to the APA, PN should be used only in extreme cases when oral and enteral forms of nutrition delivery have been attempted and failed or if the patient has a nonfunctional GI tract (GRADE 1C recommendations) [12]. Parenteral nutrition carries significant inherent risks and lacks evidence for improving ARFID-related feeding behaviors [56].

Studies on the use of nutrition support therapies in this population are heterogeneous, with variable outcomes reported. Although involving a pediatric population, Dovey et al. suggest that the use of EN or PN in the treatment of ARFID may worsen fear of oral intake and reinforce avoidance/cautious behavior [57]. Cooper et al. studied underweight patients with ARFID and anorexia nervosa enrolled in a meal-based inpatient behavior treatment program and found that the use of enteral or parenteral nutrition prior to admission to inpatient treatment was associated with a lower rate of weight gain and a greater frequency of reported GI complaints, including nausea, abdominal pain, and early satiety [17]. This suggests that pre-treatment reliance on nutrition support may signal greater illness severity or contribute to GI symptom amplification, potentially impairing subsequent weight restoration. In studying patients with ARFID receiving multidisciplinary treatment, Bern et al. showed no difference in weight change on the basis of whether or not EN was used [58]. Recommendations regarding when to begin EN are also highly variable, with one report recommending initiating EN for patients achieving less than 2 pounds of weight gain per week [59], while another concluding that lack of weight gain indicated that treatment plan modifications are needed rather than EN initiation [60].

There are potential risks of inappropriate parenteral nutrition use in this challenging patient]. Complications of PN are well-established, and its use is associated with significant long-term morbidity, including catheter-associated complications, metabolic complications, and adverse effects on quality of life for both patients and their caregivers. A recent study found that PN-dependent patients have reduced life expectancy, with greater than 17 years of average life lost compared to the general population [61]. Pathologizing functional or dysautonomia-related GI symptoms has the potential to lead to overuse of invasive nutrition therapies, placing patients at increased risk of these known PN complications. This is highly relevant for the adult ARFID population, given, as previously mentioned, the overlapping GI symptoms (e.g., early satiety, nausea, and abdominal pain) that also occur in motility disorders and dysautonomias. Overall, there is no clear consensus that use of EN or PN is beneficial in this population, except perhaps in extreme circumstances where nutritional stabilization is urgently needed. A summary of nutrition support options, indications, risks, and benefits can be found in Table 3.

Of note, the current evidence base is limited by reliance on retrospective designs, pediatric cohorts, heterogeneous diagnostic criteria, and variable outcome measures, which may introduce bias and limit the generalizability to adult GI populations. Accordingly, many recommendations reflect expert consensus rather than high-quality prospective data. Given the limited evidence, selection of adults with ARFID for nutrition support therapy should mainly be considered in cases of severe malnutrition, ongoing weight loss, or medical instability, when oral intake remains insufficient despite optimized GI symptom management. It is the authors’ opinion that the choice to pursue nutrition support in this patient population should be made in conjunction with seeking a higher level of disordered eating care, such as through a structured nutrition rehabilitation program, when possible. To mitigate the risk of reinforcing avoidant/restrictive behaviors, initiation of nutrition support should occur within a clearly defined treatment framework that includes explicit goals, predetermined criteria for weaning, and a parallel plan for oral intake advancement. Again, PN should be reserved for rare circumstances in which the GI tract is nonfunctional or when other nutrition support therapy interventions have failed despite multidisciplinary involvement.

## 7. Future Directions and Research Gaps

A number of research gaps exist for adults with ARFID who have overlapping GI disorders. The shared and distinct pathophysiological mechanisms between ARFID and GI disorders are poorly understood. Clarifying whether GI symptoms act primarily as precipitants, perpetuating factors, or consequences of ARFID will require well-designed longitudinal cohort studies that follow patients across disease trajectories and treatment exposures. In addition, evidence-based treatments for ARFID in adults are lacking. Most interventions are adapted from pediatric populations or general eating disorder protocols, and there is a lack of randomized controlled trials evaluating integrated behavioral and medical approaches in this group. Such randomized trials of pharmacological and nonpharmacological interventions, including the use of various forms of nutrition support, are needed. Additionally, more data, such as through pragmatic clinical trials, on the long-term medical, nutritional, and psychosocial outcomes of adults with ARFID and GI comorbidity are needed. Validation of ARFID screening tools in GI populations also represents a key area for future inquiry. Finally, the impact of ARFID on disease course, quality of life, and healthcare utilization in GI disease populations requires further study.

## 8. Conclusions

ARFID is a complex and underrecognized condition at the intersection of psychiatry, gastroenterology, and nutrition. In adults, particularly those with GI comorbidities, ARFID can contribute to a vicious cycle of food avoidance, malnutrition, and worsening GI symptoms, often leading to psychosocial and medical/nutrition consequences. Nutrition support therapies, including oral supplementation, enteral nutrition, and, in rare cases, parenteral nutrition, can play a critical role in stabilizing nutritional status while the underlying issues are being investigated and treated; however, their optimal use in adults with ARFID remains poorly defined, and complications are to be expected. When GI disorders and ARFID overlap, effective treatment requires an individualized, multidisciplinary approach that addresses both the psychological and physiological aspects of the disorder, ideally involving collaboration between gastroenterologists, dietitians, mental health providers, and other specialists. Despite the growing recognition of ARFID in adult populations, particularly in GI settings, significant research gaps persist. Future studies are needed to clarify the underlying mechanisms, validate screening instruments in the GI population, define evidence-based treatment protocols, and evaluate long-term outcomes. Finally, establishing clear clinical guidelines and performing high-quality research will be essential to improving care for this vulnerable population that is increasingly being encountered in clinical practice.

## Figures and Tables

**Table 1:** APA DSM-5-TR ARFID presentations and core clinical features.

ARFID Presentation (DSM-5-TR)	Defining Features	Common Clinical Manifestations
Sensory-Based Avoidance	Avoidance of foods due to sensory qualities (taste, texture, smell, appearance); food neophobia.	Limited variety; preference for bland or “safe” foods; strong aversion to mixed textures; rigid food preferences beginning in childhood or adulthood.
Fear of Aversive Consequences	Restriction driven by fear of negative consequences of eating (e.g., choking, vomiting, abdominal pain).	Reduction in intake following triggering event (e.g., choking episode, severe vomiting, food impaction, painful GI flare).
Apparent Lack of Interest in Eating/Low Appetite	Persistent low appetite, early satiety, indifference toward food, or low motivation to eat.	Slow eating, low hunger cues, inadequate caloric intake, unintentional weight loss; may be associated with neurodevelopmental or psychiatric comorbidities.
Associated Impairment	Any presentation results in: (1) significant weight loss/faltering growth; (2) nutritional deficiency; (3) dependence on supplements/EN/PN; (4) psychosocial impairment.	Varies by subtype; may involve medical instability, micronutrient deficiencies, or inability to participate in meals or social activities.

**Table 2:** Selected symptomatic therapies to address common gastrointestinal symptoms.

Symptom Targeted	Therapy/ Intervention	Mechanism/ Notes	Key Considerations in ARFID/DGBI	References
Bloating/ Distention	Low-FODMAP diet with reintroduction	Reduces fermentable carbohydrate intake; improves bloating and QOL in IBS and FD	Only under trained gastroenterology dietitian supervision; assess eating disorder risk factors; discontinue if not beneficial	[7,8,38]
Bloating/ Distention, Global GI Symptoms	Brain–gut behavioral therapies (CBT, gut-directed hypnotherapy)	Modulates visceral hypersensitivity, reduces GI-specific anxiety, improves QOL	Shown to reduce both ARFID fear (52%) and GI-specific anxiety (42%); safe and complementary to other treatments; can be delivered in as few as 4–8 sessions	[38,39,40]
Abdominal Pain/ Cramping	Antispasmodics, peppermint oil	Smooth muscle relaxation, visceral analgesia	Ranked second for pain relief in IBS; minimal nutritional or ARFID risk	[41]
Constipation	Secretagogues (linaclotide, lubiprostone, tenapanor)	Increases intestinal fluid secretion, improves colonic transit	Linaclotide ranked first for abdominal pain in IBS-C; FDA-approved for IBS-C/CIC; safe in ARFID/DGBI overlap; dose-dependent diarrhea	[8,40,41]
Constipation/Gastroparesis	Prokinetics (prucalopride)	5-HT4 agonist; accelerates gastric emptying and colonic transit	Preferred over erythromycin due to cardiac safety in patients susceptible to electrolyte abnormalities; FDA-approved for chronic idiopathic constipation	[8,42]
All Presentations	Routine diet monitoring and eating-disorder-risk counseling	Prevents iatrogenic ARFID development, supports nutritional adequacy	Essential in all DGBI patients; markers include weight loss, inadequate intake, psychological distress around eating, inflexible attitudes toward dietary expansion	[7,8]

**Table 3:** Nutrition support therapies in adults with ARFID: indications, benefits, and limitations.

Nutrition Support Modality	Description/Indications	Potential Benefits	Key Limitations and Risks	Role in ARFID
Oral nutrition support	First-line approach	Preserves oral feeding; supports gradual diet expansion; improves nutritional adequacy; aligns with ARFID behavioral treatment	May be insufficient in severe malnutrition; requires engagement and multidisciplinary support	Preferred initial strategy for most adults with ARFID
Enteral nutrition (EN)	Considered when oral intake is inadequate or patient is medically unstable	Allows rapid caloric delivery; may stabilize acute malnutrition	Minimal impact on normalization of eating; risk of reinforcing avoidance; tube discomfort, reflux, epistaxis; potential dependence	Temporary, time-limited rescue therapy only in selected cases
Parenteral nutrition (PN)	Reserved for nonfunctional GI tract or failure of oral/enteral nutrition	Bypasses GI tract; may stabilize life-threatening malnutrition	Risk of catheter-related infections, metabolic complications, reduced quality of life, and increased mortality; no evidence of ARFID symptom improvement	Strongly discouraged except in extreme, medically necessary situations
Prolonged invasive nutrition support	Long-term EN or PN use	None demonstrated for ARFID outcomes	Reinforces fear-based restriction; increases morbidity; risk of mislabeling functional symptoms as “intestinal failure”	Avoid whenever possible

## Data Availability

No unique data was generated for this review.
